# Does an 8-week home-based exercise program affect physical capacity, quality of life, sick leave, and use of psychotropic drugs in patients with pulmonary embolism? Study protocol for a multicenter randomized clinical trial

**DOI:** 10.1186/s13063-017-1939-y

**Published:** 2017-05-30

**Authors:** Nanna Rolving, Barbara C. Brocki, Hanne R. Mikkelsen, Pernille Ravn, Jannie Rhod Bloch-Nielsen, Lars Frost

**Affiliations:** 1 University Clinic of Innovative Patient Pathways, Diagnostic Centre, Silkeborg Regional Hospital, Silkeborg, Denmark; 2 Department of Occupational Therapy and Physiotherapy, Aalborg University Hospital, Aalborg, Denmark; 3 Department of Cardiology, Silkeborg Regional Hospital, Silkeborg, Denmark; 4 Department of Physiotherapy and Occupational Therapy, Silkeborg Regional Hospital, Silkeborg, Denmark; 5 Institute of Clinical Medicine, Aarhus University, Aarhus, Denmark

**Keywords:** Pulmonary embolism, Exercise, Study protocol, Randomized clinical trial, Multicenter study

## Abstract

**Background:**

The existing evidence base in pulmonary embolism (PE) is primarily focused on diagnostic methods, medical treatment, and prognosis. Only a few studies have investigated how everyday life is affected by PE, although many patients are negatively affected both physically and emotionally after hospital discharge. Currently, no documented rehabilitation options are available for these patients. We aim to examine whether an 8-week home-based exercise intervention can influence physical capacity, quality of life, sick leave, and use of psychotropic drugs in patients medically treated for PE.

**Methods:**

One hundred forty patients with incident first-time PE will be recruited in five hospitals. After inclusion, patients will be randomly allocated to either the control group, receiving usual care, or the intervention group, who will be exposed to an 8-week home-based exercise program in addition to usual care. The intervention includes an initial individual exercise planning session with a physiotherapist, leading to a recommended exercise program of a minimum of three weekly training sessions of 30–60 minutes’ duration. The patients have regular telephone contact with the physiotherapist during the 8-week program. At the time of inclusion, after 2 months, and after 6 months, the patients’ physical capacity is measured using the Incremental Shuttle Walk test. Furthermore the patients’ quality of life, sick leave, and use of psychotropic drugs is measured using self-reported questionnaires. In both randomization arms, all follow-up measurements and visits will take place at the hospital from which the patient was discharged. Levels of eligibility, consent, adherence, and retention will be used as indicators of study feasibility.

**Discussion:**

We expect that the home-based exercise program will improve the physical capacity and quality of life for the patients in the intervention group. The study will furthermore contribute significantly to the limited knowledge about the optimal rehabilitation of PE patients, and may thereby form the basis of future recommendations in this field.

**Trial registration:**

ClinicalTrials.gov, NCT02684721. Registered on 20 January 2016.

**Electronic supplementary material:**

The online version of this article (doi:10.1186/s13063-017-1939-y) contains supplementary material, which is available to authorized users.

## Background

Pulmonary embolism (PE) is a condition associated with substantial morbidity and mortality [1, 2]. In Northern Europe, there is an estimated incidence of about 100 per 100,000 person-years, with incidence rates increasing with age for both genders [1, 2]. The 30-day mortality is reported to be as high as 10–30%, depending on the size and localization of the thrombosis [3]. These numbers correspond well with incidence and mortality rates reported in Denmark, the 2012 incidence of PE being 90 and 100 per 100,000 for women and men, respectively, accumulating to a total of 2779 incidents in 2012 [4]. For those who survive a PE event, physical wellbeing and quality of life are significantly reduced [5–8], and it has furthermore been found that younger patients have a significantly higher use of psychotropic drugs up to 5 years after their PE event [9]. In Denmark, the typical patient pathway following discharge after acute PE does not address these negative consequences of PE, as health care contact is limited to a few outpatient visits during the first year after discharge, with a main focus on monitoring and adjusting anticoagulant medication. Unlike patients with myocardial infarction, who receive a 12-week rehabilitation program as standard care in Denmark, no rehabilitation is offered for patients with PE. The European guidelines on management of acute PE similarly have no recommendations regarding rehabilitation after PE [10], even though early mobilization has been shown to be safe [11]. A retrospective cohort study correspondingly found no activity-related adverse events in 422 patients with PE participating in a 3-week inpatient rehabilitation program with various activities (e.g., Nordic walking, chair exercises, ergometer bicycle training) [12]. However, although safety of early physical activity has been proven, a randomized trial is necessary to determine the effectiveness of a rehabilitation program for patients with acute PE. Here, one American RCT was found, examining the effect of a 3-month exercise and behavioral weight program on body mass index (BMI), physical activity, and cardiorespiratory fitness (*V*0_2peak_) [13]. However, only 19 patients were included, of whom 17 completed the study, making any conclusions difficult. Furthermore, no patient-reported outcome measures (PROM) were used (e.g., quality of life, disease impact on everyday life, sick leave). Thus, we lack knowledge of whether participation in a physical rehabilitation intervention can improve self-reported physical and mental well-being for this group of patients. If we look toward the fields of cardiac rehabilitation or chronic obstructive pulmonary disease (COPD) rehabilitation, there is well-founded evidence that physical exercise has a significant positive effect on quality of life, physical capacity, fatigue, and dyspnea [14, 15].

The aim of our study is therefore to investigate the effect of an 8-week home-based exercise intervention on physical capacity, quality of life, sick leave, and use of psychotropic drugs in patients with PE. We hypothesize that patients participating in the home-based exercise program will have significantly improved physical capacity and quality of life, and correspondingly reduced sick leave and use of psychotropic drugs compared to patients who receive usual care. The trial is a parallel-group, multicenter, randomized clinical superiority study with 6 months’ follow-up, including 140 patients with PE randomized in a 1:1 ratio to either control or experimental treatment.

## Methods

### Study design

**Fig. 1:**
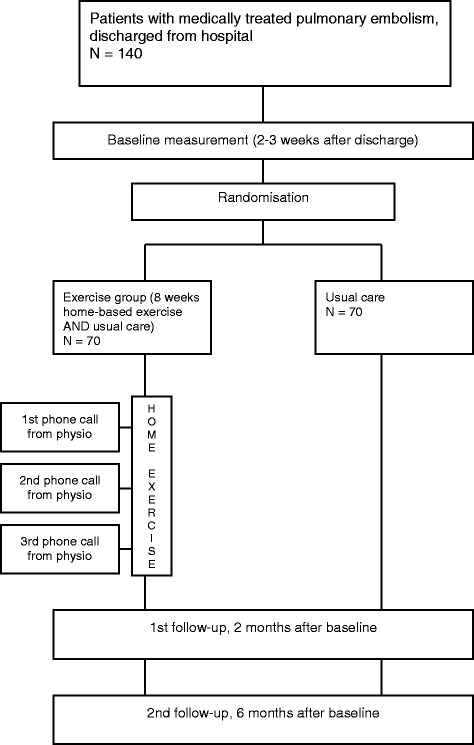
Flowchart showing the patient flow through the study

The study is a multicenter, randomized, controlled, assessor-blinded, superiority trial, investigating the effect of a low-cost home-based exercise program commenced after hospitalization for acute pulmonary embolism and continued for 8 weeks at home. The expected flow of patients through the trial can be seen in Fig. 1.

### Study setting

Recruitment of patients takes place at four regional hospitals (Silkeborg, Viborg, Horsens, and Herning) and one university hospital (Aalborg) (see ClinicalTrials.gov for contact information of the individual study sites). Table 1 provides an overview of the trial characteristics.

**Table 1:** Trial registration data

Data category	Information
Primary registry and trial identification number	ClinicalTrials.gov: NCT02684721
Date of registration in primary registry	20 January 2016
Secondary identifying numbers	The Ethics Committee of Central Denmark Region: 1-10-72-243-15 The Danish Data Protection Agency: 1-16-02-494-15
Source(s) of monetary or material support	Trygfonden (grant number ID: 115586) Diagnostic Centre, Regional Hospital Silkeborg
Primary sponsor	Trygfonden
Secondary sponsor	Diagnostic Centre, Regional Hospital Silkeborg
Contact for public queries	NR (Nanna.Rolving@midt.rm.dk)
Contact for scientific queries	NR, LF, BCB
Public title	The effect of a home-based exercise program after pulmonary embolism
Scientific title	Does an 8-week home-based exercise program affect physical capacity, quality of life, sick leave and use of psychotropic drugs in patients with pulmonary embolism? A multicenter randomized clinical trial
Country of recruitment	Denmark
Health condition(s) or problem(s) studied	Home-based exercise after pulmonary embolism
Interventions	Intervention: 8-week home-based exercise program guided by physiotherapist after discharge from hospital. Control: usual care
Key inclusion and exclusion criteria	Inclusion criteria: incident primary pulmonary embolism; treatment with anticoagulant drugs; 18–80 years of age; competency in the Danish language. Exclusion criteria: pulmonary embolism secondary to other disease; severe co-morbidity; inability to perform Shuttle Walk test; pregnancy
Study type	Interventional Allocation: randomized Blinding: assessor and investigator blind
Date of first enrolment	May 2016
Target sample size	140
Recruitment status	Recruiting
Primary outcome(s)	Incremental Shuttle Walk test Time frame: change from baseline to 6 months after inclusion
Key secondary outcome(s)	Quality of life (Pulmonary Embolism Quality of Life questionnaire) Sick leave (number of days in a period of 4 weeks) Use of psychotropic drugs (average weekly intake in a period of 2 weeks)

### Eligibility criteria

The inclusion criteria are as follows: (1) objectively verified acute PE (not recurrent PE); (2) treatment of PE with anticoagulant drugs; (3) 18–80 years of age; and (4) competency in the Danish language. Patients will be excluded in the case of: (1) PE as a secondary finding in relation to a scan performed due to another disease; (2) severe co-morbidity (e.g., severe heart disease—NYHA class 4, severe COPD—GOLD class D, active cancer, severe psychiatric disease); (3) inability to perform the Incremental Shuttle Walk test (e.g., due to amputation or intermittent claudication); (4) pregnancy; and (5) prescription of a structured rehabilitation plan to the patient’s home municipality from the discharging hospital (which entails up to 20 sessions of publicly paid, supervised physical rehabilitation within a limited time frame).

### Recruitment procedure and assignment of intervention

On the day of discharge, the nurses or physiotherapists attending the ward inform eligible patients about the ongoing study both verbally and in writing. If required, the patients are given 2 days’ deliberation time before they decide on participation in the study. The patients accepting participation are subsequently called in 2 to 3 weeks after discharge for completion of a written consent form, baseline tests, and questionnaires. Following the baseline tests, the patients are randomly allocated to either the control group or the exercise group by the use of opaque, sealed envelopes. Generation of the allocation sequence is handled by the primary investigator (PI) (NR), who is not otherwise involved in the practical enrollment and assignment of patients to the two interventions. Block randomization (by hospital) is used to ensure an equal allocation to intervention and control group at each recruitment site. The enrollment and actual randomization procedure is handled by the physiotherapist performing the baseline measurement at the hospital from which the patient was discharged.

### Blinding

Due to the nature of the intervention, the patients cannot be blinded to the allocation group. The physiotherapist performing the Incremental Shuttle Walk test (ISWT) at follow-up (assessor) is blinded to the patients’ group allocation. The PI performing the data analysis is furthermore blinded to the patients’ group allocation, as the patients’ allocation group is not provided on questionnaires or the ISWT test results. At each recruitment location, the physiotherapists performing baseline measurements and allocation keep a secured record of the included patients and their group allocation.

### Interventions

#### Control group (usual care)

Patients in the control group receive usual care. This includes zero to several days of hospitalization, depending on severity of the PE. The patient and his or her relatives receive general information about the disease and the course of treatment, the medication, and future prevention of embolism. Up to 1 year following discharge, the patient is seen on an outpatient basis for adjustment of anticoagulant treatment as required.

#### Exercise group

Patients in the intervention group receive the same usual care as patients in the control group. In addition, the patients participate in an 8-week home-based exercise program. Patients are recommended to exercise for a minimum of three times per week for 30–60 minutes, and with three to four intervals of approximately 1 minute at a high intensity level, according to the Borg CR10 Scale level 7 (only very short sentences are possible) [16]. The patients can choose whatever type of exercise they prefer, and they are generally encouraged to choose an exercise modality they already perform, or a modality they have previously had positive experiences with. Progression of the exercise program is guided by the physiotherapist at follow-up telephone calls to the patient after 1 week, 2 weeks, and 4 weeks. The aim of the physiotherapist guidance is both to keep up the patients’ motivation for exercising and to ensure the progression of the exercise program in terms of reaching a sufficiently high intensity during the interval phases during each exercise session. The patients record all their training sessions in a diary handed out at the first visit (type of exercise, duration, frequency, intensity, and additional remarks). This information will be helpful for the physiotherapist guiding the patient during the follow-up telephone calls, and furthermore aims to increase adherence to the exercise program. No intervention is offered after the 2-month follow-up, and patients are encouraged to maintain an active lifestyle.

### Concomitant care

Any form of concomitant care is allowed during the study period, reflecting usual care for this group of patients in Denmark.

### Outcome measures

#### Primary outcome measure

Physical capacity from baseline to 6-month follow-up, as measured with the Incremental Shuttle Walk test.

#### Secondary outcome measures

Quality of life measured with the PE Quality of Life (PEmb-QoL) and the EuroQol Five Dimensions (EQ-5D) questionnaires; sick leave (number of days in the last 4 weeks) and use of psychotropic drugs (type of drug, doses, and frequency in the last 4 weeks).

The Incremental Shuttle Walk test (ISWT) has been developed for assessing a person’s maximum walking capacity. It has been found to be valid, reliable, and responsive in several studies within a number of study populations, including patients with heart and lung diseases [17]. Peripheral oxygen saturation is registered at each level and patients rate their Borg CR10 dyspnea before and immediately after the test [18]. Reference values have been published for both healthy individuals and for patients with COPD and cardiac disease [19].

The Pulmonary Embolism Quality of Life (PEmb-QoL) is a disease-specific quality of life questionnaire, developed for patients with PE [20]. The PEmb-QoL questionnaire contains six dimensions, these being frequency of complaints, activities of daily living (ADL) limitations, work-related problems, social limitations, intensity of complaints, and emotional complaints. Scores for all dimensions are calculated by the sum of the scores for each item of the dimension divided by the number of the items. Questions 1, 4, 5, and 9 are reverse scored. Questions 2 and 3 provide descriptive information. Higher scores indicate worse outcome.

The EuroQol Five Dimensions (EQ-5D) is a generic quality of life questionnaire comprising five dimensions (mobility, self-care, usual activities, pain/discomfort, and anxiety/depression). Each dimension has three levels (no problems, some problems, extreme problems), resulting in a total of 245 potential health states. The scores fall on a scale of -0.624 to 1.0 (perfect health), including the scores of -0.293 for “unconscious” and 0.000 for “dead”. The instrument has been validated in Danish, including the construction of Danish preference values [21, 22].

Sick leave: the patient is asked to state the number of days’ sick leave within the last 4 weeks according to the following categories: 0 work days; 1–4 days per week; 5–7 days per week.

Use of psychotropic drugs: the patient is asked to state the type and dose of drug(s) used and their average weekly use within the last 4 weeks according to the following categories: 0 days per week; 1–4 days per week; and 5–7 days per week.

### Data collection

Data on age, gender, BMI, medical treatment of PE, type of embolism (central or peripheral), length of hospital stay, and any comorbidity are retrieved from the patient’s medical records, and are recorded on a paper sheet. Data on quality of life, sick leave days, use of psychotropic drugs, and exercise adherence are collected using self-reported questionnaires. Data on physical capacity are collected through a physical test, handled by a physiotherapist. The result is recorded on a paper sheet. All data will be collected by the physiotherapists performing the baseline and follow-up tests at the individual study sites. Data are stored at secure locations according to the requirements of the Danish Data Protection Agency.

**Fig. 2:**
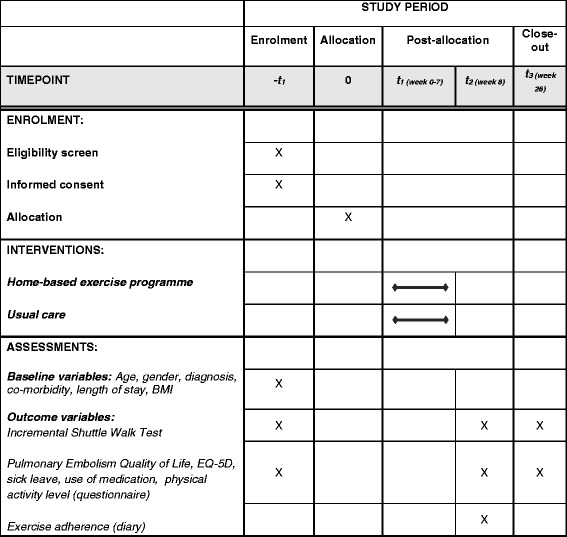
Schedule of enrollment, interventions, and outcome assessments

Data are collected at the baseline visit; data on exercise adherence are collected only at the 2-month follow-up (for patients in the exercise group); data from questionnaires and the ISWT are collected at baseline, after 2 months, and after 6 months. The two follow-up measurements are handled by a different physiotherapist, who is blinded to each patient’s treatment allocation. It is emphasized to the patients that they should take care not to disclose their group allocation at the follow-up tests. See Fig. 2 for an overview of the study period with time intervals for enrollment, interventions, and outcome assessments.

### Data management and analysis

Study data will be collected and managed using REDCap electronic data capture tools hosted at the Department of Clinical Medicine, Aarhus University [23]. STATA 13.0 (Stata Corp, College Station, TX, USA) will be used for data analysis. Data entry and analysis will be handled by the PI, who is blinded to treatment allocation. Double entry of data will be performed to ensure data quality, and range checks for data values will be performed before data analysis. For the primary outcome, change in ISWT from baseline to 6-month follow-up, parametric statistical methods will be applied for description and analysis if the data are normally distributed. For the secondary outcomes, change in PEmb-QoL and EQ-5D from baseline to 6 months, parametric or nonparametric statistical methods will be used, depending on the data distribution. Sick leave and use of psychotropic drugs will be analyzed using a chi-squared test due to the categorical distribution. Furthermore, a complementary per protocol analysis will be performed for patients complying with minimum 75% of the exercise program, to evaluate the efficacy of the intervention.

### Sample size calculation

For the ISWT, a minimum clinically relevant difference has not been established for a PE population. In a COPD population it is considered to be between 48 meters [24] and for a cardiac population it has been set to 70 meters [25]. As we consider the PE population to resemble a cardiac population more than a COPD population, a difference of 70 meters (SD = 139) [17] was chosen. A sample size of 62 patients in each group is required at a significance level of 5% and a power of 80%. Conservatively assuming a 60% recruitment rate and an 85% retention rate (taking into consideration attrition due to death and loss to follow-up), we would need to approach 243 patients to achieve a sample of 146 at pretest and 124 at post-test (i.e., 62 per group). Analysis of data will be performed according to intention-to-treat principles.

### Study principles

The protocol follows the SPIRIT 2013 checklist provided in (Additional file 1) (Standard Protocol Items: Recommendations for Interventional Trials) [26], whereas the reporting of the study will follow the CONSORT Statement (Consolidated Standards of Reporting Trials), using the extension for nonpharmacological trials [27].

## Discussion

The study is expected to contribute significantly to our scant knowledge regarding physical and mental recovery of survivors of a PE event. As yet, only one small RCT including 19 patients with PE has been performed, examining the short-term effect of an exercise and diet intervention on BMI and physical capacity [13]. The present study aims to examine the effect of a home-based exercise intervention on both physical capacity and patient-reported outcome measures. When designing the intervention, it was as important to make it a simple, low-cost intervention in order to gain maximal patient adherence to the exercise program, and at the same time make it realistic to implement in our health care system subsequently. Should the study find the expected improvement in physical capacity, quality of life, sick leave, and use of psychotropic drugs for the patients participating in the exercise intervention, it may lead to new recommendations regarding the rehabilitation of patients with PE.

### Data monitoring

No data committee has been established as the intervention is considered to be low risk. All patients and physiotherapists will be asked to report any adverse events to the primary investigator, and the trial will be stopped if any adverse events are considered to have been caused by the exercise intervention or the physical test. The authors meet frequently during the study to discuss trial conduct.

### Trial status

Recruitment of patients was initiated by centers on 1 May 2016, and the last center began inclusion early September 2016. As of April 1st 2017, 60 patients are included in the study. With an expected inclusion rate of seven to eight patients per month in total, an estimated recruitment period of 18–20 months is required, counting from the initiation of recruitment in May 2016.

### Dissemination of results

The results of the study will be shared with the public and with peers through publication in peer-reviewed journals and in the public press, and through presentations at conferences.

## Additional file

Additional file 1: Spirit checklist. (DOC 122 kb)

## Abbreviations

BMI: Body mass index
COPD: Chronic obstructive pulmonary disease
EQ-5D: EuroQol Five Dimensions questionnaire
ISWT: Incremental Shuttle Walk test
PE: Pulmonary embolism
PEmb-QoL: Pulmonary Embolism Quality of Life questionnaire
PI: Primary investigator
